# Differential profiling of lacrimal cytokines in patients suffering from thyroid-associated orbitopathy

**DOI:** 10.1038/s41598-018-29113-2

**Published:** 2018-07-17

**Authors:** Edina Kishazi, Marianne Dor, Simone Eperon, Aurélie Oberic, Natacha Turck, Mehrad Hamedani

**Affiliations:** 1 OPTICS Group, Department of Human Protein Science, University of Geneva, Geneva, Switzerland; 2 Department of Ophthalmology, University of Lausanne, Jules-Gonin Eye Hospital, Fondation Asile des Aveugles, Lausanne, Switzerland

## Abstract

The aim was to investigate the levels of cytokines and soluble IL-6R in the tears of patients with thyroid-associated orbitopathy (TAO) disease. Schirmer’s test was adopted to collect tears from TAO patients (N = 20, 17 women, mean age (±SD): 46.0 years (±13.4)) and healthy subjects (N = 18, 10 women, 45.4 years (±18.7)). Lacrimal cytokines and soluble IL-6R (sIL-6R) were measured using a 10-plex panel (Meso Scale Discovery Company) and Invitrogen Human sIL-6R Elisa kit, respectively. Tear levels of IL-10, IL-12p70, IL-13, IL-6 and TNF-α appeared significantly higher in TAO patients than in healthy subjects. Interestingly, IL-10, IL-12p70 and IL-8 levels increased in tears whatever the form of TAO whereas IL-13, IL-6 and TNF-α levels were significantly elevated in inflammatory TAO patients, meaning with a clinical score activity (CAS) ≥ 3, compared to controls. Furthermore, only 3 cytokines were strongly positively correlated with CAS (IL-13 Spearman coeff. r: 0.703, p = 0.0005; IL-6 r: 0.553, p = 0.011; IL-8 r: 0.618, p = 0.004, respectively). Finally, tobacco use disturbed the levels of several cytokines, especially in patient suffering of TAO. The differential profile of lacrimal cytokines could be useful for the diagnosis of TAO patients. Nevertheless, the tobacco use of these patients should be taken into account in the interpretation of the cytokine levels.

## Introduction

Thyroid-associated orbitopathy (TAO) is an autoimmune disorder, very often related to Graves’ disease. Clinical manifestations are mainly proptosis (eye protrusion), lid retraction and eye movement limitation with diplopia. During the acute phase, there are some inflammatory signs such as lid edema, conjunctival edema (chemosis), redness of the eye or lids^1,2^. These clinical signs permit the scoring of the inflammatory process (Clinical Activity Score, CAS) and the monitoring of patients under anti-inflammatory treatment^3^. Smoking is considered as a main risk factor for TAO disease. It has been shown that patients with more severe eye disease are more likely to be smokers, than those with less severe or no disease^4^.

The diagnosis of Graves’ disease is based on the detection of circulating auto-antibodies against thyroid stimulating hormone receptor (TSHR) in patient’s blood. Concerning the orbitopathy, there is no specific biomarker available at the moment.

TAO may occur (5–10% of cases) before any thyroid dysfunction and without any detectable auto-antibodies^2^. That is why a specific biomarker of the orbital disease would be very useful for the diagnosis and the follow-up of the patients.

Tears could be a relevant source of biomarker because of the proximity of the inflammatory process and the frequent involvement of lacrimal gland in TAO^5^. Furthermore, collection of tears is easy and non-invasive (Schirmer’s paper).

Despite the progress done in the understanding of TAO disease, some important pieces are still missing. Nevertheless, the target cells seem to be the fibroblasts presenting the thyroid stimulating hormone receptor (TSHR)^6–9^. They display surface receptors for cytokines what render them as potential target for the immune system. Several cytokines have been shown to be present in serum^10–14^, in orbital fat and muscles^15–17^, and tears^18,19^ of patients suffering from TAO. Cytokines could be used as potential biomarkers since they are identified as major actors in the TAO disease.

The goal of our study is to measure the levels of 10 cytokines (Interferon- γ (IFN-γ), interleukins IL-10, IL-12-p70, IL-13, IL-1β, IL-2, IL-4, IL-6, IL-8, Tumor necrosis factor-α (TNF-α) and soluble Interleukin-6 receptor (sIL-6R)) in TAO patients’ tears compared to control subjects. Potential correlations between the levels of these cytokines and the clinical features of TAO patients were also investigated. Considering that smoking has a negative impact on the evolution of TAO, we finally observed its potential influence on cytokines and sIL-6R.

## Results

Our study included 20 TAO patients (17 women) with a mean age of 46.0 years (±13.4) and 18 healthy subjects (10 women) with a mean age of 45.4 years (±18.7). No significant difference was observed between age and gender of these 2 groups. 14 out of 20 TAO patients had a more active form of the disease (with a CAS ≥ 3). 9 TAO patients were active tobacco consumers at the time of the tear collection apart from one person, who stopped a couple of weeks before, but was still considered as a smoker. Among control subjects, 6 were active tobacco consumers. The demographic and clinical data describing TAO and control patients are presented in Table 1.

**Table 1:** Demographic description of the study cohort.

	Controls	TAO	p-value*
Gender (M/F)	8/10	3/17	0.074
Age (mean ± SD) years	45.4 ± 18.7	46.0 ± 13.4	0.939
Eye (Left/Right)	8/10	10/10	0.757
Smokers	6	9	0.522
CAS	N.A		N.A.
0		2	
1		1	
2		3	
3		4	
4		5	
5		5	

The number of TAO patients showing a clinical activity score (CAS) from 0 to 5 are indicated. N.A: Not Applicable; M: male/F: female; SD: Standard deviation; CAS: Clinical Activity Score; *Fischer tests for categorical variables and Mann-Whitney U tests for continuous variables.

### Cytokines and sIL-6R concentrations in tears of TAO and control patients

**Figure 1:**
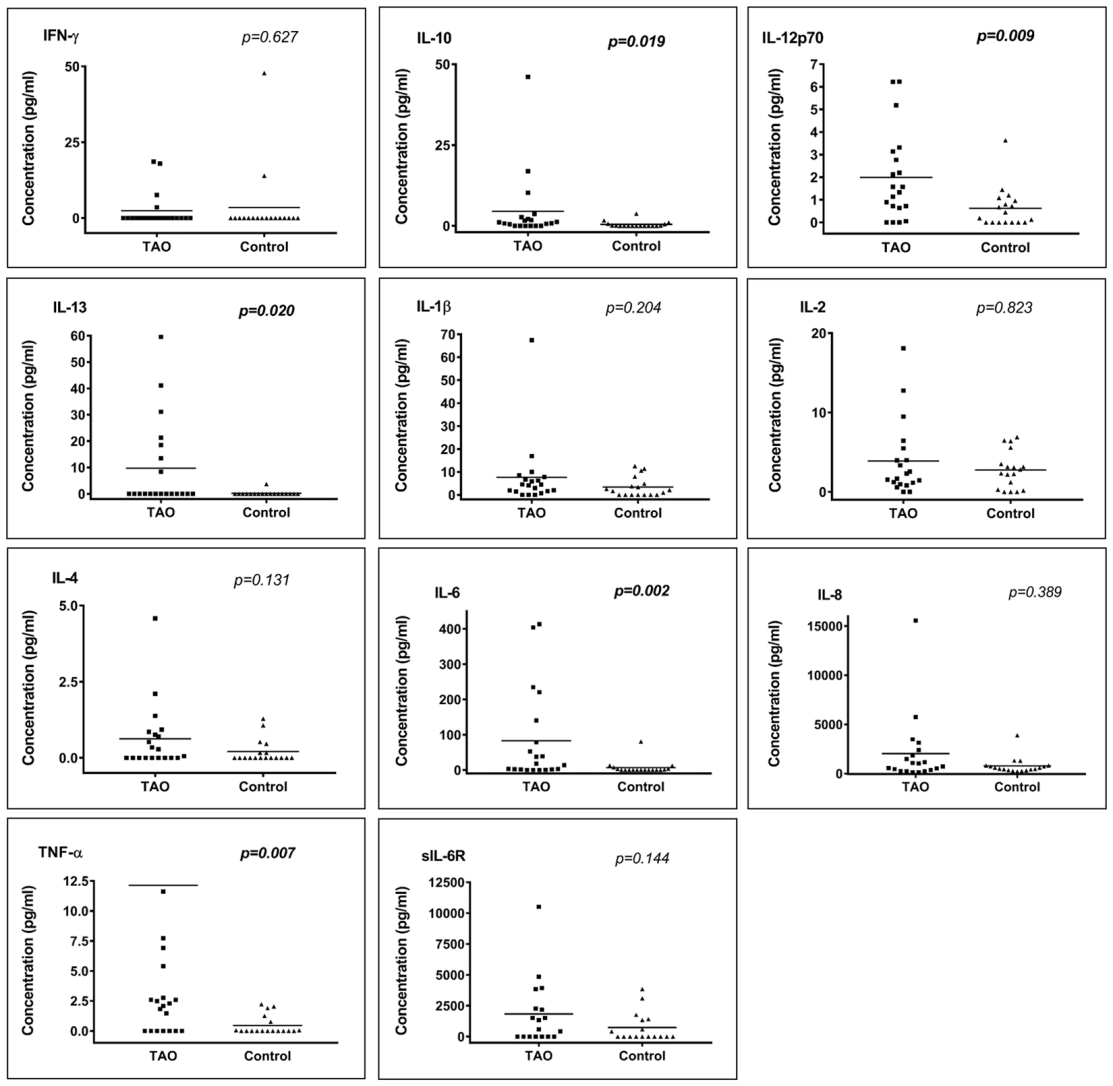
Cytokine concentrations in tears of TAO patients versus those of control patients (pg/ml). *Mann-Whitney U tests, p-values (bold data highlight significant p-values). For esthetical reason, the point of one TAO patient was removed for TNF-α, as he had a concentration much higher than the other patients (193.20 pg/ml).

The lacrimal concentration of 10 cytokines and sIL6-R in TAO patients and control subjects are shown in Fig. 1 (details of the cytokine concentrations can be found in Supplementary Information S1). All molecules tended to be more concentrated in TAO patients than in control patients, except for IFN-γ, for which control tears showed a slightly more elevated level. The concentrations of 5 cytokines (IL-10, IL-12p70, IL-13, IL-6 and TNF-α) appeared significantly higher in TAO patients than in control subjects.

**Figure 2:**
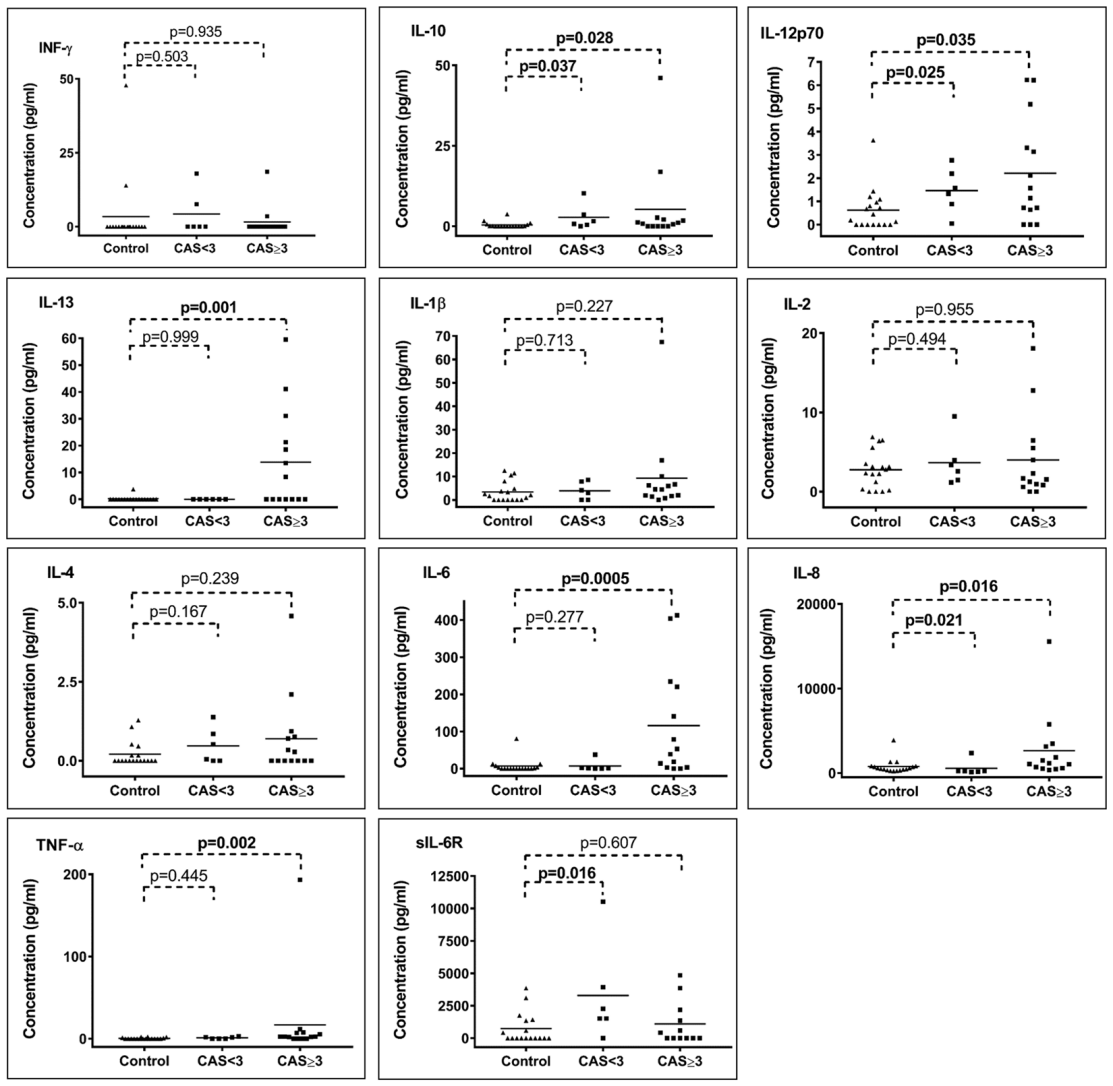
Cytokine concentrations (pg/ml) in tears of control subjects, patients showing a CAS < 3 and patients showing a CAS ≥ 3. *Mann-Whitney U tests, p-values (bold data highlight significant p-values).

Detailed analyses showed that IL-10, IL-12p70 and IL-8 levels were significantly elevated whatever the form of TAO (CAS < 3 and CAS ≥ 3, Fig. 2 and Supplementary Information S2). The IL-13, IL-6 and TNF-α were significantly elevated in TAO patients with CAS ≥ 3 compared to controls. Nevertheless, none of the cytokines was able to distinguish between patients with mild form of TAO and control subjects. At the opposite, sIL6- R levels could discriminate patients with mild form of TAO and control subjects (p = 0.016) but not patients with more severe form of TAO and control patients.

### Tear cytokine concentrations correlation with CAS in TAO patients

**Figure 3:**
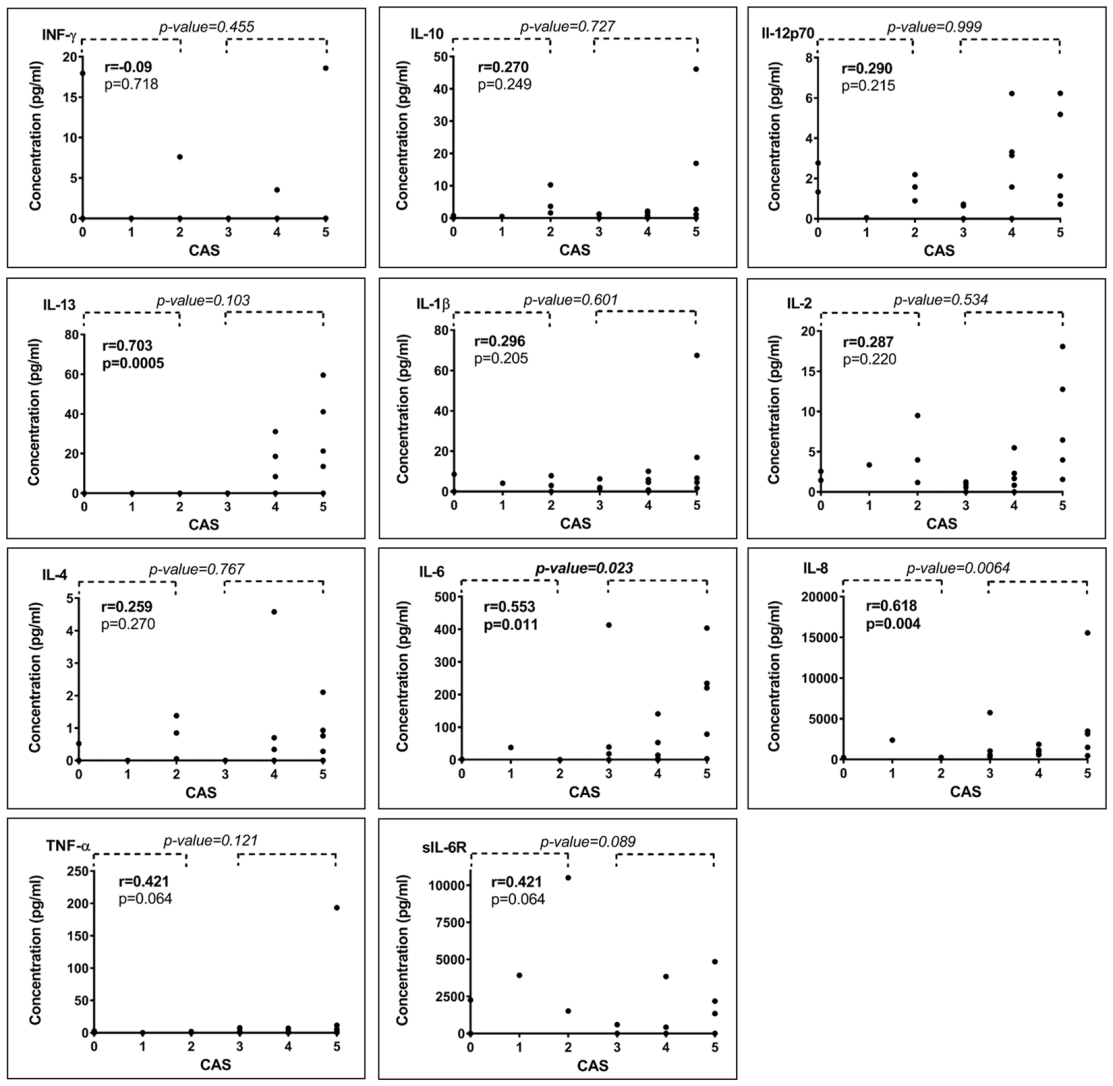
Cytokine concentration (pg/ml) in tears of TAO patients according to their CAS. P-values on the top of the figure relates to comparison between patients presenting CAS ≥ 3 and patients presenting CAS < 3. On the left of the figure, Spearman correlation (r) and its p-value are indicated.

Cytokine concentrations were therefore evaluated according to their potential relationship with the activity of the disease (Fig. 3). The IL-13, IL-6 and IL-8 cytokines were strongly positively correlated with the activity of the disease (CAS) (Spearman coeff. r: 0.703, p = 0.0005; r: 0.553, p = 0.011; 0.618, p = 0.004, respectively). Furthermore, we dichotomised patients according to their CAS (moderate to severe inflammatory form when CAS ≥ 3 and mild inflammatory form for CAS < 3) and compared their cytokine concentrations in tears (Fig. 3). The group with a CAS ≥ 3 showed a significant up-regulation in the concentration of IL-6 (p-value = 0.023) and IL-8 (p-value = 0.006).

### Tear cytokine concentrations and smoking status in TAO and control patients

**Figure 4:**
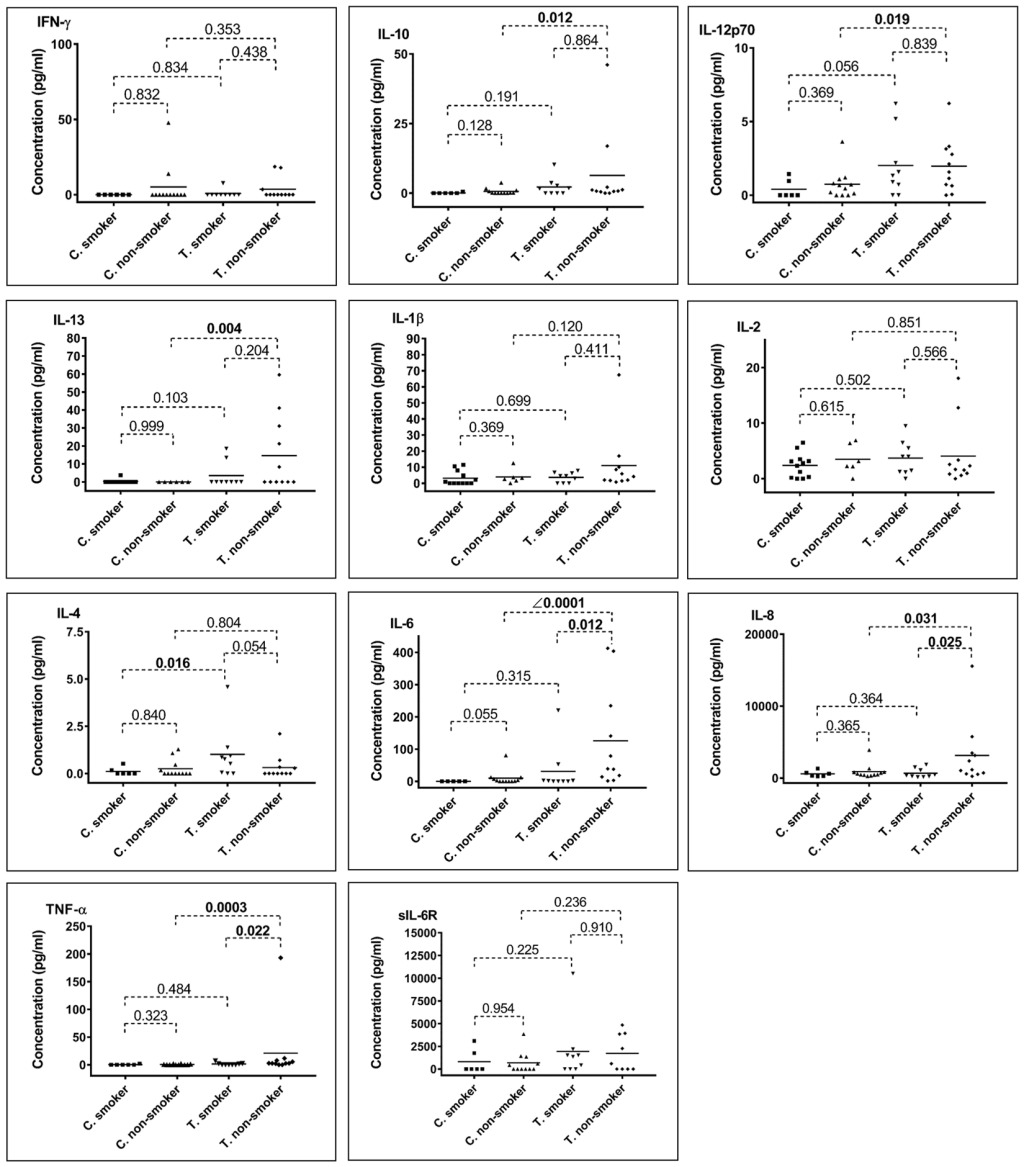
Cytokine concentrations (pg/ml) in tears of TAO patients (T) and control subjects (C), comparing subgroups of smokers and non-smokers. *Mann-Whitney U tests, p-values (bold data highlight significant p-value).

We investigated tear cytokine concentrations in patient’s grouping according to their smoking status (smokers *vs*. non-smokers after grouping patients according to their pathology). In control subjects, no significant difference was observed between non-smokers and smokers control subjects (details can be found in Supplementary Information S3). On the opposite, in tears of TAO patients, 3 cytokines exhibited significantly more elevated levels in non-smoker patients (Fig. 4 and Supplementary Information S3) compared to smokers (IL-6: p-value = 0.012, IL-8: p-value = 0.025, TNF-α: p-value = 0.022). Then, we grouped all subjects according to their tobacco use (Supplementary Information S4). Tears of TAO smokers presented higher levels of IL-4 compared to tears of control smokers (p = 0.016, Supplementary Information S4). Furthermore, in the non-smokers group, IL-10, IL-12p70, IL-13, IL-6, IL-8 and TNF-α were significantly increased in TAO patients versus control non-smokers.

## Discussion

Thyroid-associated orbitopathy (TAO) is the common ‘extra-thyroid’ manifestation of Graves’ disease. This is an autoimmune disorder in which specific auto-antibodies bind and target orbital fibroblasts that release chemokines leading to infiltration of lymphocytes. Through a variety of mechanisms, activated orbital fibroblasts produce therefore significant inflammation, oedema and fat deposition resulting in increased orbital volume and therefore characteristic eye protrusion (exophthalmos or proptosis) usually observed in TAO disease.

In this context, the general mechanism leading to the pathological event has been largely elucidated, with a key place occupied by inflammatory cytokines and chemokines. Investigating their levels in tears of TAO patients could be therefore particularly informative of the clinical status of the TAO patients. Indeed, tears emerge as promising fluid for the discovery of biomarkers and for better understanding diseases, especially for those which could lead to ocular manifestations. The relatively low volume available after collection is counter-balanced by high protein concentration and the sensitivity of multiplexing ELISA. Several studies on ocular or associated diseases have already demonstrated the presence of elevated inflammatory cytokines in tears^5,18–22^. Furthermore, as lacrimal glands can be affected by TAO disease^5,23,24^, tears could be a central actor, but not deeply studied yet in this context.

The regulation of the inflammation, an important process of the TAO disease, is subtly mediated by cytokines and chemokines that can be naively classified into pro and anti- inflammatory components. Nevertheless, several of them present pleiotropic functions depending on stimuli or environment. The 10-plex chosen to investigate the tears of TAO patients contained a mix of pro and anti-inflammatory cytokines. Our study showed net lacrimal elevation of these cytokines in TAO patients compared to healthy subjects, but only the increases of IL-10, IL-12p70, IL-6, IL-13 and TNF-α were significant. Furthermore, IL-12p70, IL-10 and IL8 levels were higher whatever the subtypes of TAO considered and IL-6, IL-13 and TNF-α seemed to be more expressed in patients presenting the more active phase of TAO.

Globally, our findings obtained for TNF-α, IL-6, IL-8 IL-10 and IL-13 were completely relevant of TAO and confirmed previous studies on tears^5,18,19^ but also on serum^19–22,25^. They also confirmed some results that we obtained performing quantitative proteomics analyses with mass spectrometry^26^. Our major result was that two proteins related to the inflammation, cystatin C and serpin A3, were found significantly upregulated in tears of TAO patients compared to controls (ratio TAO/controls = 1.53 for cystatin and 1.7 for serpinA3). This is fully in line with the cytokine level increases that we observed in the current article between TAO patients and healthy controls. Indeed, IL-6 is one of the major regulator of serpinA3^27^, found as a stimulus of the synthesis and secretion of this protein^28^. Concerning cystatin C, TNF-α was shown to upregulate its expression while IL-10 is supposed to decrease it^29^. The fact that cystatin C was found upregulated despite an upregulation of IL-10 may imply that the upregulation power of the TNF-α is greater than the downregulation power of IL-10 on the cystatin C expression. This duality may also be the reason why cystatin C was found with a lower upregulated ratio in TAO patients (1.53) compared to serpinA3 (1.7). The global conclusion of the two parts of our study is that the inflammatory process appeared as the main deregulated pathway in TAO disease and could be, both at cytokine and protein levels, a very promising target for better management of the patients.

In addition, TNF-α, IL-6, IL-8 IL-10 and IL-13 have already been described for various involvements in TAO. These include (i) stimulation of the glycosaminoglycan production *in vitro* in ocular fibroblast for TNF-α^30,31^, (ii) secretion by orbital fibroblasts under the stimulation of TSHR antibodies or prostaglandin E2 for IL-6^32–34^, (iii) expression by fibrocytes that can induce the infiltration of inflammatory cells and subsequent tissue remodelling for IL-8^35^ and the steroid treatment which decreases its expression level for IL-8^22^ and (iv) regulation of both T and B-cells functions for IL-10^36^. Finally, for all of them, genetic polymorphism of their gene in relation with TAO have been identified in different populations^21,37–44^.

By comparison with the previous cytokines described, IL-12p70 (shortly called IL-12) is few investigated in TAO to date. Nevertheless, its expression is induced by TSHR and CD40^45,46^. Serum elevation of IL-12^47^ and polymorphism association have been reported in TAO patients^45^.

Interestingly, TNF-α, IL-12p70, IFN-γ and IL-2 belong to the cytokines T Helper 1 (Th1) that mediate the development of organ-specific autoimmune disease and are considered as positive effectors of inflammation. However, in our study, IFN-γ and IL-2 did not display the same pattern as IL-12p70 and TNF-α. No significant variation was observed for them in our study. Even if these results are surprising regarding the potential role of IFN-γ in the pathogenesis of TAO, similar results were nevertheless previously reported in blood^48–50^. The low percentage of samples above the limit of detection could be another explanation. Concerning IL-2, controversial data exist^18,20,21,51,52^ and at this stage, our data cannot confirm or dismiss these preliminary findings.

At the opposite, IL-10, IL-13 and IL-6 are produced by Th2 cells. The involvement of both CD4+ T cell populations is described in TAO disease^53–56^. Their predominance seems tissue-specific: Th1 cells are more detected in extracellular muscles whereas Th2 cells are located preferably in orbital tissues. Moreover, their importance seems to be course-dependent: Th1 cells are present in active phase and Th2 in inactive phase^57^.

Smoking has been shown to greatly influence TAO^58,59^. As expected we observed disturbed levels of some cytokines in patients suffering from this disease. The TNF-α, IL-6, IL-8 cytokines seemed particularly affected by the smoking status of patients. They appear significantly down-regulated in smokers as compared to non-smokers. The recording of this information is therefore crucial in the management of the TAO patients and can be taken into consideration for the interpretation of the biological parameters in these patients. This result raises also a point that remains too often ignored concerning the influence of interfering factors on cytokine measurements. A steroid effect has been speculated^60–63^ without being clearly studied and could be particularly pertinent in ocular diseases in which steroids treatment is often administrated. Other factors could be interesting to investigate including patient parameter (such as age^64–66^, physical activity^67–69^, diet^70–72^) but also pre-analytical sampling (time of collection, storage, stability in different biological fluids)^73,74^ and even co-existing diseases. That may become even more likely if tears are considered as the biological fluid because not so much information are also available concerning its variability and a lot of studies remain to be done for establishing all the knowledge required for its routine clinical use. This point has to be solved by robust multi-centric studies before proposing to daily use of cytokines measurements. In addition, as no standardisation procedure exists, multiple bioassays or immunoassays platforms (Luminex, Mescoscale, single array, microarray) have been also used for quantifying cytokines. This technological variability induces therefore different sensitivity, specificity, accuracy and makes therefore very difficult comparison between the few studies existing in tears. Indeed, the dynamic range of concentration reported for control subjects are different from one study to another^5,18,19^ and ours is not an exception. This can also explain why some data appear controversial in the cytokines studies.

Two other studies from Huang *et al*.^5,18^ and one from Ujhelyi *et al*.^19^ also measured some cytokines in the context of TAO diseases, in similar cohorts of patients. While we confirmed some of their results (upregulation of TNF-α, IL-6 and IL-13 in TAO patients), our work permitted to highlight IL 12-p70 as a promising cytokine but also some non-concordant results between their studies and our (for IL-1β, IL-2 and IL-8). Moreover, by taking into account the smocking statue, we opened the question of how this parameter needs to be considered in clinical investigations. All these points clearly established the need to deeper explore the cytokine question for TAO disease.

Some limitations in this investigation have to be mentioned. The sample size is relatively small, probably masking significant results. It is nevertheless in the traditional size of cohort reported for this disease. Considering the sample size of our subgroups (smocking status and CAS), we are aware that these data have to be carefully taken into account. However, it constitutes a first step to go deeper in the investigation of TAO disease. In addition to the size, only few but crucial clinical information has been collected, limiting the possibility to go deeper in the analyses (for example correlation between the levels of thyroid antibodies and cytokines cannot be studied). Introducing in the future studies, a group of Graves’ disease without orbitopathy could be also clinically pertinent. Regarding the technical part, we were limited by the sensitivity of the ELISA kits. It leaded to more or less high percentages of out of detection values for both TAO patients (from 0% of out of detection values for IL-8 to 75% for IFN-γ; mean = 30%) and controls (from 0% for IL-8 to 94% for IL-13; mean = 52%). However, we observed that in general we have a higher percentage of out of range for controls compared to TAO patients, which could be related to the fact that all the 11 molecules seemed to be in lower concentration among controls.

Overall, this study indicated that (i) elevated levels of cytokines can be observed in TAO patients and (ii) correlation exist with the activity of the disease but their levels could be influenced by the smoking status. Nevertheless, even if their use for TAO diagnosis or prognosis remains purely speculative at this stage, the observation of their elevations, decrease or stable levels could help to better understand this complex disease and therefore better manage patients.

## Methods

### Sample collection

Subjects > 18 years old were recruited at the Jules-Gonin Eye Hospital (Lausanne, Switzerland) between December 2013 and July 2015. Written informed consent was obtained from all subjects and the study was carried out according to the ethical standards (Declaration of Helsinki). The cantonal ethics committee for research on human beings has approved the patient’s informed consent form and the use of biological material (Human Research Ethics Committee in Lausanne, No 204/14, July 2014, Annex 2). We included 20 TAO patients who did not suffer from any other ocular pathology and 18 control subjects who did not suffer from any systemic or ocular pathology, or suffered from a benign pathology such as ptosis or blepharochalasis. Assessing classical tests used in ophthalmology, patients with dry eye syndrome were excluded from the two groups to avoid any effect of ocular surface disorder on our results^75^. The Dry Eye WorkShop (2007)^76^ advice a cut-off value of ≤ 5 mm in 5 minutes in order to diagnosis dry eye subjects. All our patients gave more than 5 mm wetting in 3 minutes so we reduced the collection time to 3 minutes. In addition to the Schirmer test, dry eye diagnosis was done using ocular surface fluorescein staining method, following classical protocol. Patients wearing lenses and pregnant women were also excluded. Among the collected samples, 10 out of the 18 controls and 6 out of the 20 TAO patients were also used in the proteomics study that we carried in parallel^26^.

A sheet form was filled for each patient, summarising the demographic data and recording its medical history, both general and ocular anamnesis and smoking status. Indeed, since smoking negatively affects the course and severity of TAO, we differentiated between non-smokers and smokers.

To measure the inflammatory activity of the disease^1,77^, we recorded, for each eye, the clinical activity score (CAS), which is the sum of 7 items present; spontaneous retrobulbar pain, pain on attempted up or down gaze, swelling of the eyelids, redness of the eyelids, redness of the conjunctiva, conjunctival oedema and inflammation of the caruncle. The CAS cut-off at 3 discriminates between the mild inflammatory form of the disease and the moderate to severe form of the disease. A CAS ≥ 3 suggests initiating an immunosuppressive treatment.

Schirmer paper strips (Biotech Vision Care PVT LTD, Gujarat, India) were placed inside the lower eyelid of patient’s both eyes during a maximum of 3 minutes. A mean wetting of 19.5 ± 4.3 mm (range: 13–30 mm) was obtained in 3 min for healthy control subjects and 19.1 ± 5.9 mm (range: 10–30 mm) for TAO patients. No anaesthetics or eye drops were used before sample collection. After collection, the strip was inserted in a tube on ice and centrifuged at 7840 g for 7 min at 4 °C without any additional buffer, as described elsewhere^78,79^, and then immediately frozen at −80 °C. Both eyes were collected for each subject but were not pooled, and only one out of the two was used for further analyses. Concerning the eye selection, the main criterion was the CAS. We tried to have a majority of CAS ≥ 3, however when the two eyes of a TAO patient had a CAS < 3, we chose the eye with the higher CAS for further analyses. For controls, eye selection was done in order to homogenise both age and sex according to the TAO patient selection.

### Cytokine measurement

Cytokine measurement was done using the multiplex Human Pro-inflammatory Panel 1 from Meso Scale Discovery company (lot: K0080448, catalogue number: K15049-D1, Rockville, MD, USA). The multiplex ELISA kit contains Interferon- γ (IFN-γ), IL-10, IL-12p70, IL-13, IL-1β, IL-2, IL-4, IL-6, IL-8, Tumor necrosis- α (TNF-α) spotted per well as a sandwich ELISA. Regarding the user manual, median lower limit of detection were the following: IFN-γ = 0.2 pg/ml, IL-10 = 0.03 pg/ml, IL-12p70 = 0.11 pg/ml, IL-13 = 0.24 pg/ml, IL-1β = 0.04 pg/ml, IL-2 = 0.09 pg/ml, IL-4 = 0.02 pg/ml, IL-6 = 0.06 pg/ml, IL-8 = 0.04 pg/ml and TNF-α = 0.04 pg/ml. Minor changes were made to the original protocol provided by manufacturer, as follows: tear samples were diluted 50 times, limited volumes (25 µl instead of 50 µl) of samples were added in each well of the 96-well plate and incubated 2 hours at room temperature. After 3 washing steps, incubation with the capture antibody was carried out 2 hours at room temperature followed by an overnight incubation at 4 °C in order to improve the sensitivity of the assay. The plate was washed then 3 times and the read buffer containing electroluminescent SULFO-TAG labelled detection antibody was added. The 96-well plate was read on MSD Sector Imager (Rockville, MD, USA) which measures the light emitted upon applied voltage. The data were analysed with Discovery Workbench 4.0. software (Rockville, MD, USA).

### Soluble Interleukin-6 receptor (sIL-6R) measurement

Soluble IL-6R was measured using Invitrogen Human sIL-6R Elisa kit (supplied by Thermofischer, Catalogue number: #KHR0061, Waltham, MA, USA), which is a colorimetric assay. Regarding the user manual, the analytical sensitivity of the kit was <8 pg/ml. The assay was carried out according to the manufacturer’s instructions, except that tears were diluted 50 times and incubation with the capture antibody was carried out overnight. The plate was read on Filtermax F3 Multi-mode microplate reader (Molecular Devices, Sunnyvale CA, USA), and the data were analysed by Softmax Pro Microplate reader (Molecular Devices, Sunnyvale, CA, USA) and the associated- analysis software (version 6.2).

For cytokine and sIL-6R measurements, samples were tested in duplicate using 1 µl of sample per well. Means of the calculated concentrations were used for comparison and statistical analyses and the coefficient of variation was in the expected range of the assays.

### Statistics

Statistical analysis and graphs were carried out in GraphPad Prism (version 7.02, GraphPad Software, Inc., La Jolla, CA, USA). As the distribution of the data didn’t follow Gaussian distribution, nonparametric Mann-Whitney U-test was used for 2 group comparisons (or Kruskal-Wallis test if more groups were considered). A two-tailed p-value smaller than 0.05 was considered as significant. Spearman correlation coefficient between cytokine concentration and CAS was also calculated using this software. For all cytokines, out of range values were set as “zero”, in order to perform statistical analyses without losing information.

### Data availability statement

The datasets generated during and/or analysed during the current study are available from the corresponding author on reasonable request.

## Electronic supplementary material

Supplementary information
