# Supplementary material for: Differential profiling of lacrimal cytokines in patients suffering from thyroid-associated orbitopathy
Source: Sci Rep. 2018 Jul 17;8:10792. doi: 10.1038/s41598-018-29113-2 (PMC6050228; doi:10.1038/s41598-018-29113-2)
Supplement: Supplementary file 1 — Supplementary information [file 41598_2018_29113_MOESM1_ESM.pdf]

# Differential profiling of lacrimal cytokines in patients suffering from thyroid-associated orbitopathy

Edina Kishazi[1], Marianne Dor[1], Simone Eperon[2], Aurélie Oberic[2], Natacha Turck[1*], Mehrad Hamedani[2*]

| Cytokine concentration Mean ± SD (pg/ml) | Controls (N=18) | TAO (N=20) | p-value* |
|---|---|---|---|
| IFN-γ | 3.43±11.55 | 2.38±5.74 | 0.627 |
| **IL-10** | **0.43±0.94** | **4.50±10.63** | **0.019** |
| **IL12-p70** | **0.63±0.89** | **1.99±1.96** | **0.009** |
| **IL-13** | **0.21±0.88** | **9.67±16.83** | **0.020** |
| IL-1β | 3.43±4.30 | 7.68±14.67 | 0.204 |
| IL-2 | 2.75±2.32 | 3.89±4.68 | 0.823 |
| IL-4 | 0.20±0.39 | 0.62±1.09 | 0.131 |
| **IL-6** | **6.71±18.95** | **83.17±132.0** | **0.002** |
| IL-8 | 777.98±841.55 | 2025.75±3488.20 | 0.389 |
| **TNF-α** | **0.46±0.80** | **2.61±3.19** | **0.007** |
| sIL-6R | 735.29±1191.56 | 1833.33±2665.41 | 0.144 |

Supplementary information S1: Cytokine concentrations in tears of TAO and control patients. *: Mann-Whitney U tests, p-values (bold data highlight significant p-values)**;** SD: standard deviation.

| Cytokine concentration (pg/ml) Mean ± SD | Controls | CAS<3 | p-value* | CAS≥3 | p-value† |
|---|---|---|---|---|---|
| IFN-γ | 3.43±11.55 | 4.26±7.37 | 0.503 | 1.58±4.99 | 0.935 |
| **IL-10** | **0.43±0.94** | **2.77±3.88** | **0.037** | **5.24±12.55** | **0.028** |
| **IL12-p70** | **0.63±0.89** | **1.47±0.96** | **0.025** | **2.21±2.26** | **0.035** |
| IL-13 | 0.21±0.88 | 0±0 | 0.999 | **13.82±18.77** | **0.001** |
| IL-1β | 3.43±4.30 | 3.91±3.70 | 0.713 | 9.29±17.32 | 0.227 |
| IL-2 | 2.75±2.32 | 3.66±3.05 | 0.494 | 3.99±5.32 | 0.955 |
| IL-4 | 0.20±0.39 | 0.47±0.56 | 0.167 | 0.69±1.27 | 0.239 |
| **IL-6** | 6.71±18.95 | 7.1±14.88 | 0.277 | **115.77±146.85** | **0.0005** |
| **IL-8** | **777.98±841.55** | **561.62±889.50** | **0.021** | **2653.23±4008.19** | **0.014** |
| **TNF-α** | 0.46±0.80 | 1.01±1.18 | 0.445 | **16.92±50.85** | **0.002** |
| **sIL-6R** | **735.29±1191.56** | **3291.67±3762.96** | **0.016** | 1104.14±1674.27 | 0.607 |

Supplementary information S2: Cytokine concentrations (pg/ml) in tears of control subjects, patients showing a CAS<3 and those showing a CAS≥ 3**.** *: Comparison between tears of control subjects and tears of TAO patients showing a CAS< 3, Mann-Whitney U tests, p-value (bold data highlight significant p-values); † comparison between tears of control subjects and tears of patients showing a CAS≥3, Mann-Whitney U tests, p-value (bold data highlight significant p-values).

| Cytokine concentration (pg/ml) mean±SD | TAO | | | Control | | |
|---|---|---|---|---|---|---|
| | Non-smokers | Smokers | p-value* | Non-smokers | Smokers | p-value* |
| IFN-γ | 3.64±7.31 | 0.85±2.54 | 0.438 | 5.14±14.02 | 0±0 | 0.838 |
| IL-10 | 6.37±14.05 | 2.21±3.30 | 0.864 | 0.61±1.11 | 0.08±0.20 | 0.128 |
| IL12-p70 | 1.97±1.84 | 2.01±2.22 | 0.839 | 0.74±1.00 | 0.40±0.64 | 0.369 |
| IL-13 | 14.6±20.87 | 3.56±7.18 | 0.204 | 0.31±1.08 | 0.00±0.00 | 0.999 |
| IL-1β | 10.98±19.35 | 3.64±3.07 | 0.411 | 3.16±4.43 | 3.98±4.40 | 0.369 |
| IL-2 | 4.06±5.82 | 3.69±3.08 | 0.566 | 2.38±2.16 | 3.48±2.67 | 0.615 |
| IL-4 | 0.31±0.64 | 1.01±1.42 | 0.054 | 0.25±0.46 | 0.11±0.21 | 0.840 |
| **IL-6** | **125.78±165.22** | **31.08±72.98** | **0.012** | 10.07±22.76 | 0.00±0.00 | 0.055 |
| **IL-8** | **3138.45±4445.23** | **665.78±644.04** | **0.025** | 880.14±991.78 | 573.67±410.89 | 0.365 |
| **TNF-α** | **20.94±57.23** | **1.39±2.27** | **0.022** | 0.51±0.82 | 0.34±0.83 | 0.323 |
| sIL-6R | 1721.30±2019.85 | 1945.37±3314.94 | 0.910 | 809.72±1324.22 | 694.70±1178.88 | 0.954 |

Supplementary information S3: Cytokine concentrations (pg/ml) in tears of TAO and control patients, each group being divided according to patient smoking status. *: Mann-Whitney p-values (bold data highlight significant p-values); SD: Standard Deviation.

| Cytokine concentration (pg/ml) mean±SD | Non-smokers | | | Smokers | | |
|---|---|---|---|---|---|---|
| | TAO | Control | p-value* | TAO | Control | p-value* |
| **IFN-γ** | 3.64±7.31 | 5.14±14.02 | 0.353 | 0.85±2.54 | 0±0 | 0.843 |
| **IL-10** | **6.37±14.05** | **0.61±1.11** | **0.012** | 2.21±3.30 | 0.08±0.20 | 0.191 |
| **IL-12p70** | **1.97±1.84** | **0.74±1.00** | **0.019** | 2.01±2.22 | 0.40±0.64 | 0.056 |
| **IL-13** | **14.6±20.87** | **0.31±1.08** | **0.004** | 3.56±7.18 | 0.00±0.00 | 0.103 |
| **IL-1β** | 10.98±19.35 | 3.16±4.43 | 0.120 | 3.64±3.07 | 3.98±4.40 | 0.669 |
| **IL-2** | 4.06±5.82 | 2.38±2.16 | 0.851 | 3.69±3.08 | 3.48±2.67 | 0.502 |
| **IL-4** | 0.31±0.64 | 0.25±0.46 | 0.804 | **1.01±1.42** | **0.11±0.21** | **0.016** |
| **IL-6** | **125.78±165.22** | **10.07±22.76** | **<0.0001** | 31.08±72.98 | 0.00±0.00 | 0.315 |
| **IL-8** | **3138.45±4445.23** | **880.14±991.78** | **0.031** | 665.78±644.04 | 573.67±410.89 | 0.364 |
| **TNF-α** | **20.94±57.23** | **0.51±0.82** | **0.0003** | 1.39±2.27 | 0.34±0.83 | 0.484 |
| **sIL-6R** | 1721.30±2019.85 | 694.70±1178.88 | 0.236 | 1945.37±3314.94 | 809.72±1324.22 | 0.225 |

Supplementary information S4: Cytokine concentrations (pg/ml) in tears of non-smokers on one side and smokers on the other side. *: In each group, values are compared between TAO patients and controls. Mann-Whitney p-values (bold data highlight significant p-values); SD: Standard Deviation.
